# A-I-D for cascades: an application of the Behaviour Change Wheel to design a theory-based intervention for addressing prescribing cascades in primary care

**DOI:** 10.1186/s43058-024-00673-x

**Published:** 2024-12-05

**Authors:** Lisa M. McCarthy, Barbara J. Farrell, Colleen Metge, Lianne Jeffs, Sameera Toenjes, M. Christine Rodriguez

**Affiliations:** 1 Leslie Dan Faculty of Pharmacy, University of Toronto, Toronto, ON Canada; 2 Institute for Better Health, Trillium Health Partners, Mississauga, ON Canada; 3 Bruyère Health Research Institute, Ottawa, ON Canada; 4 Women’s College Hospital, Toronto, ON Canada; 5 Department of Family Medicine, University of Ottawa, Ottawa, ON Canada; 6 Department of Community Health Sciences, Max Rady College of Medicine, University of Manitoba, Winnipeg, MB Canada; 7 Science of Care Institute and Lunenfeld-Tanenbaum Research Institute, Sinai Health, Toronto, ON Canada; 8 Institute of Health Policy, Management and Evaluation, University of Toronto, Toronto, ON Canada

**Keywords:** Behaviour change, Deprescribing, Polypharmacy, Side effects, Drug, Prescribing cascades

## Abstract

**Background:**

Prescribing cascades, which occur when a medication is used to treat the side effect of another medication, are important contributors to polypharmacy. There is an absence of studies that evaluate interventions to address them. We describe an application of the Behaviour Change Wheel (BCW) to design theory-informed interventions for addressing prescribing cascades within interprofessional primary care teams.

**Methods:**

The BCW framework was applied to guide intervention development. This report describes the first seven steps. Three behaviours were developed based on data collected from two qualitative studies exploring why and how cascades occur across practice settings. A target behaviour was selected and the COM-B model was applied to identify relevant factors for interprofessional primary care teams. Relevant intervention types, policy options, and corresponding behaviour change techniques (BCTs) were identified, and intervention examples drafted. Prioritization of behaviours and intervention examples were guided by the APEASE criteria.

**Results:**

The three behaviours involved supporting: (1) healthcare providers (HCPs) to ask about, investigate and manage cascades, (2) the public to ask about prescribing cascades, and (3) the public to share medication histories and experiences with HCPs. The team selected the HCP behaviour, A-I-D (ask, investigate, deprescribe), for intervention development. Psychological capability and physical opportunity were the most relevant COM-B components. Ten intervention options comprised of BCTs were developed, which are ready for further prioritization by stakeholders. These can be grouped into: provision of educational materials for use by HCPs; provision of consultation or training to support HCPs; and knowledge mobilization strategies. Through the process, the team identified that development of a practice guidance tool, which assists HCPs to investigate and manage prescribing cascades, is needed to support further intervention development.

**Conclusions:**

The BCW framework guided the design of intervention examples to support primary HCPs practicing in interprofessional teams to address prescribing cascades. When identifying interventions for future consultation, creation of a practice guidance tool was prioritized as it underpins all proposed interventions for addressing prescribing cascades in practice. Further research is needed to determine what primary HCPs would need in this practice guidance tool and how it will be used in practice, to support its development.

**Supplementary Information:**

The online version contains supplementary material available at 10.1186/s43058-024-00673-x.

## Contributions to the literature

- This is the first study to apply the Behaviour Change Wheel framework to design interventions to address prescribing cascades in clinical practice.
- Intervention design efforts focused on primary care interprofessional teams as they were considered to face fewer barriers for addressing prescribing cascades compared to solo practitioner primary care settings.
- Our team was comprised of scientists, pharmacists, and nurses who have researched prescribing cascades or experienced them in practice. Applying the BCW framework required that several decisions be made by the interdisciplinary team, each decision undertaken is transparently reported, adding to the study’s rigour.

## Background

Prescribing cascades are important and underrecognized contributors to problematic polypharmacy, making them a valuable target for deprescribing efforts. They occur when a medication is used to manage the adverse effects of another medication, whether intentionally or when a medication-related adverse effect is misinterpreted as a new medical condition. Prescribing cascades can be classified as appropriate or inappropriate (problematic) [1]. For a prescribing cascade to be considered ‘appropriate’, prescribing a second medication to manage an adverse event must be done so knowingly by the prescriber after an assessment of alternatives, and a determination, in collaboration with the patient, that this action is the best available option [1].

Studies using health administrative data have investigated the prevalence and harms associated with potentially inappropriate prescribing cascades [2–5]. Due to the large number of examples in practice (over 300) and emerging data on harm, there is important potential for population-level impact [6]. This is particularly true when it comes to considering the emerging data on harm. For example, the calcium channel blocker-loop diuretic cascade has been linked to increased risk of hospitalization and emergency department visits as well as reduced quality of life in terms of physical functioning [6, 7]. Further, by contributing to polypharmacy, inappropriate prescribing cascades also put individuals at a higher risk of adverse drug events, medication interactions, falls, fractures, and other medication-related harms [8].

Qualitative studies conducted by our team have explored how and why prescribing cascades occur across a variety of care settings, including ambulatory and primary care clinics, and long-term care homes [9, 10]. We found that prescribing cascades are complex and contextually situated. Further, while early publications have conceptualized addressing cascades as a linear process (i.e., prevent-identify-resolve), our results demonstrated that the processes of investigating and managing cascades are iterative and linked. Trials of deprescribing to manage cascades, can also be used as a method of investigating potential cascades.

Although tools and guidance are available to support healthcare providers’ efforts to identify and investigate prescribing cascades, they are not widely used in practice. Piggott et al. (2020) described how to apply process mapping to the investigation of cascades [11]. ThinkCascades is an internationally developed consensus-based short list of clinically important prescribing cascades that suggest potentially inappropriate prescribing for older adults [12]. Others have published questions for healthcare providers to consider when evaluating the appropriateness of a cascade [1]. Notably, there is an absence of resources to guide management (i.e., deprescribing) of prescribing cascades, and studies that evaluate the implementation and impact of existing tools and guidance, as well as other interventions to address prescribing cascades [13]. This study aims to address this important gap by applying a theory-guided approach to intervention development.

Best practice recommendations (e.g., Medical Research Council Framework) strongly suggest using theory to guide intervention development [14, 15]. The Behaviour Change Wheel (BCW) framework, centred in theory, provides a clear, systematic process for determining which behaviour change strategies are applicable to a particular context [16]. The BCW integrates the COM-B model, which analyzes the behaviour in question by considering the capability (C), opportunity (O) and motivation (M) to enact behaviour (B) change. This behavioural analysis guides the selection of potential intervention types, which are linked to evidence-based behaviour change techniques suitable for intervention design.

The BCW framework has increasingly been used by different groups to develop interventions targeted at healthcare providers, including interventions developed to address polypharmacy in the context of multimorbidity [17] and to support deprescribing in hospital and long-term care settings [18–21]; however, it has not been applied to the development of interventions to address prescribing cascades. Herein, we describe our application of the BCW framework to design an intervention(s) to address prescribing cascades at the individual, practice, and healthcare system levels.

## Methods

### Design

This is a qualitative study drawing from pre-existing interview data collected from two primary qualitative studies [9, 10].

### Source of data

In earlier studies, we conducted two series of qualitative interviews (*n* = 51) involving 18 patient cases to understand more about the phenomenon of prescribing cascades. For each case, we interviewed a patient who may have experienced at least one prescribing cascade as well as their family caregiver (where applicable) and healthcare providers the patient identified as well positioned to provide additional information. Patients were recruited from three care settings in Canada including a geriatric day hospital, long-term care, and primary care sites [10]. Our objectives were to understand why and how prescribing cascades occur, explore factors associated with their prevention, development, and management, and to identify strategies to improve healthcare providers’ ability to address them. The methods (including interview guides) and results of these qualitative studies have been published elsewhere [8, 9].

### Data analysis

We used the BCW framework, and seven of its eight steps, to guide intervention development across three stages (understand the behaviour, identify behaviour options, identify content and implementation options).

#### BCW Stage 1: understand the behaviours

##### Step 1: define the problem in behavioural terms

Transcripts from the primary studies were reviewed by the study team to identify potential behaviours that could be performed by patients, caregivers and healthcare providers to address prescribing cascades (i.e., prevent potential cascades as well as identify and manage existing cascades).

##### Step 2: select the target behaviour

It is common when identifying problems in behavioural terms to generate many ‘potential problems’ on which to focus. To select the target behaviours, the team assessed the impact of the behaviour change, ease of implementation of an intervention focused on that behaviour, potential for spill over (i.e., benefits for other behaviours), ease of measurement. Each target behaviour was assigned a global prioritization as per BCW guide (unacceptable, less promising, promising, or very promising) [22].

##### Step 3: specify the target behaviour

The team used the AACTT framework (action, actor, context, time, target) to specify the target behaviours i.e., who needed to do what differently, where and when [23]. The action refers to the behaviour that needs to change in terms of what can be observed or measured; actor is each person or people who could do each of the actions targeted; context refers to the physical location, emotional context, or social setting in which the action is performed; target is the person or people for whom the action is performed; and lastly, time refers to when the action is performed, i.e., date, time, frequency, etc.

##### Step 4: identify what needs to change to achieve the desired behaviour

The lead investigator (LM) identified the components from the COM-B (capability, opportunity, motivation-behaviour) model thought to be most influential for the selected target behaviour drawing from our prior interviews [9, 10]. These were presented to the study team for input and refinement at a consensus meeting. Guided by the COM-B model, which states that capability and opportunity are necessary precursors for motivation, the team also prioritized which COM-B components were the most important drivers of the behaviour at the consensus meeting.

#### BCW Stage 2: identify intervention options

##### Step 5: identify intervention types to achieve the desired behaviour

Each COM-B component was mapped to its relevant BCW intervention types. Within the BCW model, there are nine types of interventions (e.g., education, training, incentivization, etc.) that can support required behaviour change and the most relevant ones for a particular problem depend on the related COM-B components. The final intervention types were selected by the team using a consensus-based approach and guided by the APEASE criteria [22]. The APEASE criteria assess the acceptability, practicability, effectiveness, affordability, side effects/safety, and equity of an intervention to determine if it is appropriate for development. Acceptability assesses whether the intervention is acceptable to stakeholders. Practicability considers whether the intervention can be delivered as intended to the target population. Effectiveness and cost-effectiveness assess whether the intervention would be effective in the real world and is worth the cost. Side effects/safety looks at whether there are any negative or positive unintended consequences from the intervention. Affordability considers whether the intervention would be affordable to deliver to the target population. Finally, equity assesses whether disparities would be reduced or increased as a result of implementing the intervention [22].

##### Step 6: identify relevant policy categories

The selected intervention types were mapped onto the most relevant of seven policy-level options to help generate ideas for intervention development. The team again used the APEASE criteria to select relevant policy options.

#### BCW Stage 3: identify content and implementation options

##### Step 7: identify behaviour change techniques

Intervention types were combined with relevant policy options to select behaviour change techniques (BCTs) using Michie’s BCT taxonomy [24], a hierarchically structured compilation of BCTs used in behaviour change interventions. A BCT is an “observable, replicable, and irreducible components of an intervention that are designed to alter or redirect causal processes that regulate behavior; that is, a technique is proposed to be an “active ingredient” (e.g., feedback, self-monitoring, and reinforcement)” [24]. The initial list of identified BCTs were narrowed by eliminating those that were redundant. Remaining BCTs were reviewed at a consensus meeting where we discussed how they could be delivered, alone or in combination, to address prescribing cascades. The acceptability and practicability of each proposed intervention example (i.e., one or more BCTs) was assessed using the APEASE criteria to further refine the list. A final list of intervention examples was then created and assessed using all of the APEASE criteria.

## Results

### BCW Stage 1: understand the behaviour

#### Step 1: define the problem in behavioural terms

Through our qualitative interviews [9, 10], we found that to improve processes for preventing, investigating, and managing prescribing cascades, behaviors could focus both on healthcare providers who manage medications as well as individuals who may experience prescribing cascades and their caregivers. Specifically, three behavioural problems were identified:

- Healthcare providers and the public do not consistently ask if a sign or symptom could be caused by a drug.
- Even if healthcare providers and the public consider that signs and symptoms may be possible medication side effects, they are not able to easily access information (i.e., prescribing cascades tools and/or patient medication history) to support their investigation of a possible prescribing cascade or to develop a plan on how to manage it.
- Healthcare providers and the public have difficulty assessing the risk/benefit profile of continuing or deprescribing a medication, and therefore, struggle to strategize plans for investigating and managing a prescribing cascade.

#### Step 2: select the target behaviour

**Fig. 1:**
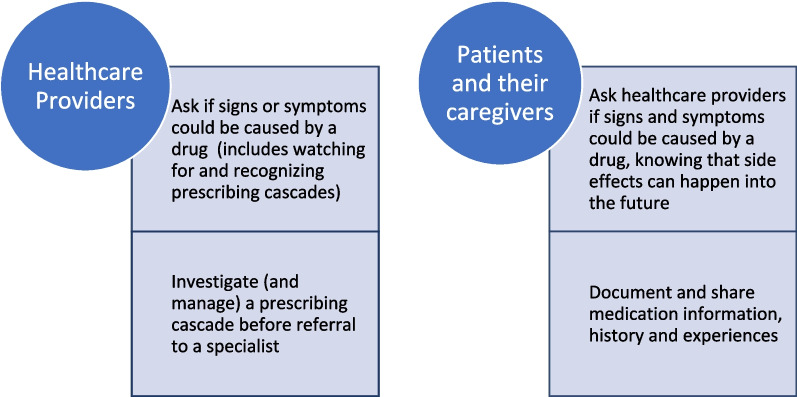
Potential Target Behaviours to Address Prescribing Cascades

We identified several target behaviours from the problems in Step 1 for further consideration (Fig. 1). Two focused on patients and their caregivers, and two were focused on healthcare providers. When reviewing the targets with the APEASE criteria (Supplementary Materials Table 1), the team debated which actor group (i.e., healthcare providers or patients and their caregivers) would be most appropriate for initial efforts. We decided to focus on healthcare providers because patient-focused targets were less promising. For the patient targets, the concern was that empowerment of patients could have the unintended consequence of frustrating healthcare providers who often lack the ability to address patient questions about prescribing cascades. Further, while supporting patients to document and share medication information, history and experience is important, an intervention focused on this behaviour would not directly impact prescribing cascades as a contributor to problematic polypharmacy.

Selecting one behaviour for intervention development is encouraged within the BCW process. However, when we tried to prioritize between the healthcare provider-focused targets, we realized that, while separate, both were necessary steps for addressing existing prescribing cascades. One cannot investigate and manage cascades without identifying potential cascades, and to identify potential cascades without continuing to act by both confirming and managing them is counter to the goal of reducing problematic polypharmacy.

#### Step 3: specify the target behaviour

Using the AACTT framework (Supplementary Materials Table 2), the healthcare provider target behaviours were further divided into three actions:

1. Ask/assess if the sign/symptom can be caused by one or more of the drugs the patient is taking.
2. Investigate the sequence of events and reasons for medications.
3. Deprescribe as appropriate.
  a To prevent a cascade or manage an existing one, you need to decrease, pause or stop potentially causative drug(s) (drug A), monitor for adverse drug withdrawal events (ADWEs).
  b Then, if a cascade exists, decrease, pause or stop drug B and monitor for ADWEs.

The target behaviours/actions were given the acronym A-I-D corresponding to ‘ask’, ‘investigate’ and ‘deprescribe’. A-I-D was classified as a multicomponent (or ‘mega’) behaviour based on our previous studies [9, 10] which found that I and D were intrinsically linked as well as analysis undertaken as part of the process described herein (not shown) that demonstrated that A-I-D as separate components had similar COM-B domains.

Relevant actors were those most likely to be involved in prescribing in our selected context, Ontario’s interprofessional primary care teams, and included physicians, nurse practitioners, nurses, and pharmacists [25]. In Ontario, primary care teams prioritize continuity of care and are able to well positioned to provide longitudinal follow up [26, 27]. When compared to solo practitioner primary care settings, such as community pharmacies or solo family medicine healthcare providers, the teams were considered to face fewer barriers for identifying, investigating and deprescribing cascades (e.g., shared electronic health records with access to the same medication and prescribing records, team relationships and communication models that facilitate discussion of complex patient cases). We excluded hospital units and long-term care due to variable involvement of some of these actors.

Our ultimate target for the behaviour, i.e., for whom the actions are performed, are patients. Time was defined when encountering a new sign/symptom or a possible prescribing cascade, including during medication reviews, before ordering tests and before referral to specialists.

#### Step 4: identify what needs to change to achieve the desired behaviour

Knowledge garnered from previous qualitative interviews informed selection of the behavioural drivers (i.e., capability, opportunity, and motivation) for the target behaviour (i.e., A-I-D) (Supplementary Materials Table 3). Overall, psychological capability and physical opportunity were deemed to be the behavioural drivers for high prioritization for intervention development for all professions. Specific to prescribing cascades, psychological capability refers to health professionals’ knowledge about medication side effects, what cascades are, their awareness of common examples of cascades, their understanding about the clinical impact of cascades, and whether they have the skills needed to investigate and deprescribe prescribing cascades [10]. Physical opportunity relates to whether health professionals have time within their daily routines and access to information sources needed to investigate prescribing cascades [9]. Social opportunity and reflective motivation were the next important behavioural drivers for prioritization. Social opportunity, in the context of A-I-D, relates to whether health professionals have support as part of an interprofessional team to discuss patient cases including possible cascades, and management approaches [10]. It also relates to whether health professionals’ feel empowered to try to deprescribe potential cascades if they were not the original prescriber of one of the medications or if they were uncertain about the reason a medication was originally prescribed [9]. We selected physical opportunity as a higher priority than social opportunity because health professionals in our past interviews emphasized the importance of time and information access as key influences on their willingness to address cascades [9, 10]. Reflective motivation impacts whether a health professional would accept accountability for investigating and potentially deprescribing medications for cascades they encounter [10].

During this process, the team determined that registered nurses were the largest outlier among the professions in terms of their clinical role in prescribing (i.e., nurses assess but lack prescribing authority in Ontario’s primary care teams at present). For this reason, physicians, nurse practitioners, and pharmacists were prioritized as actors for intervention development as they are the most involved in prescribing.

### BCW Stage 2: identify intervention options

#### Step 5: identify intervention types to achieve the desired behaviour

Given that most components of the COM-B model were considered important drivers of the target behaviour across all professions, our team faced the need to prioritize which intervention types would move forward for further intervention development. We decided that intervention types aligned solely with reflective motivation would not be considered further (i.e., persuasion, incentivization, coercion). This aligns with the COM-B model which shows that capability and opportunity are necessary precursors for motivation (i.e., when capability and opportunity are behavioural drivers, interventions that solely focus on motivation may be less impactful). The intervention types deemed most relevant for the intervention were education, training, environmental restructuring, and enablement (Table 1).

**Table 1:** Assessment of each potential intervention type using the APEASE criteria

Intervention Type	Example	COM-B Component	APEASE Summary	Retain for Intervention Development
Education	Provide information about the health consequences of missing prescribing cascades	Psychological capability	*Acceptable and practical*: Yes, for all audiences *Effective*: Unclear if effective on its own; training is more effective *Affordable*: Yes *Spillover*: Yes *Equity*: Yes, can be made available broadly	Yes
		Reflective motivation		
Training	Provide opportunities to practice the target behaviours (e.g., using mock patients)	Psychological capability	*Acceptable and practical*: Yes, may require more in-depth interactive online modules *Effective*: Yes *Affordable*: Unclear, can be costly to produce and do well *Spillover*: Yes *Equity: Yes*	Yes
		Physical opportunity		
Environmental Restructuring	Add posters or other items to prompt the healthcare provider to consider prescribing cascades before making a referral or prescribing a new medication	Physical opportunity	*Acceptable and practical*: Yes *Effective*: Potentially *Affordable*: Yes *Spillover*: Yes *Equity*: Yes	Yes
		Social opportunity		
Enablement	Create a journal club to enable regular discussion around prescribing cascades	Psychological capability	*Acceptable and practical*: Yes *Effective*: Potentially, requires uptake *Affordable*: Unclear, can be costly to build out *Spillover*: Yes *Equity*: Yes	Yes
		Physical opportunity		
		Social opportunity		
Restriction	Implementing a process whereby healthcare providers cannot make outside referrals unless prescribing cascades have been investigated	Physical opportunity	*Acceptable and practical*: No *Effective*: Potentially *Affordable*: Yes *Spillover*: No *Equity*: Yes	No
		Social opportunity		
Modelling	Use peer coaches as part of the training process	Social opportunity	*Acceptable and practical*: Potentially *Effective*: Potentially *Affordable*: Yes *Spillover*: No *Equity*: Yes	No
		Reflective motivation		

#### Step 6: identify relevant policy categories

The policy options that were deemed relevant following assessment using the APEASE criteria were guidelines, communications and marketing, and service provision (see Supplementary Materials Table 4).

### BCW Stage 3: identify content and implementation options

#### Step 7: identify behaviour change techniques

An initial list of 26 BCTs were identified and reduced to a final list of 19 BCTs (see Supplementary Materials Table 5). Intervention examples were created for as many of the 19 identified BCTs as possible, but following review, we further narrowed to 17 intervention examples (see Supplementary Materials Table 6). From this list of 17 intervention examples, 10 were selected, which were grouped into three categories: provision of educational content or materials for use of healthcare providers; provision of consultation or training to support healthcare providers; and knowledge mobilization. The final 10 interventions were assessed using all APEASE criteria (see Table 2).

**Table 2:** Final intervention examples

Intervention	APEASE Summary	Target Behaviour(s)
**Education Content**		
Good practice guidelines to help healthcare providers identify and manage prescribing cascades	*Strengths* Easily spread via knowledge mobilization efforts. Positive spillover into health professions education and would be widely available if published open access. *Weaknesses* Requires funding and time to develop.	Ask, Investigate, Deprescribe
Web-based or e-tool featuring an algorithm to help healthcare providers identify potential prescribing cascades	*Strengths* Depends on development cost but would be a helpful step forward. There is value in an implicit tool that guides people through how to tackle prescribing cascades. This could be paired with an explicit list. There is work to do to create and ‘validate’ such a tool. *Weaknesses* If needs to be interactive could be expensive (if want a ‘validated’ and frequently updated tool). Users would need online access.	Ask
Web-based or other e-tool featuring a list of prescribing cascades to help healthcare providers identify potential prescribing cascades.	*Strengths* Need to equip healthcare providers with direction on what to do next. Important to include in good practice guidelines. Easy to prepare, can post on stakeholder websites. Not expensive if static. Positive spillover in medical education, to patients, etc. Can translate into multiple languages and ensure drugs from different countries are included. *Weaknesses* There is work and expenses to create and “validate” such a tool that we would need to think through. A list may be useful for identification of cascades (not management). Challenge if claim to be comprehensive. Some have had the caveat “list is not all encompassing”. Users would need online access.	Ask
Online learning module(s) for healthcare providers to enable them to identify and manage prescribing cascades through the use of interactive case demonstrations and discussions, goal setting and action planning.	*Strengths* Considered acceptable if healthcare providers are able to get continuing education credits. Positive spillover into health professions education. *Weaknesses* This can be expensive to create, update and maintain and time-consuming to complete, although may depend on length of modules and potential registration fee or sponsor. When creating this, would need to evaluate the effectiveness and ensure it is globally accessible. Users would need online access.	Ask, Investigate, Deprescribe
Published case reports with discussion guides about prescribing cascades for interprofessional teams.	*Strengths* Can be used as a journal club or in education to stimulate discussion amongst healthcare providers about prescribing cascades. There has been prior success with deprescribing case reports published in a peer-reviewed journal (Available at: https://deprescribing.org/case-reports/). Consider the option of promoting current, existing publications and research. Open-access publications needed to optimize equitable access. *Weaknesses* Time and resources required to create and publish the cases. Knowledge mobilization efforts/partnerships would be needed to convince people to read and use these. Requires time investment from busy healthcare providers to review.	Ask, Investigate, Deprescribe
Demonstrate clinical thought process of investigating and managing prescribing cascade for healthcare providers through online resources (e.g. learning module, whiteboard video, testimonial)	*Strengths* Good uptake with deprescribing.org whiteboard videos (Available at: https://www.youtube.com/@deprescribing). Relatively inexpensive. Positive spillover to medical education. *Weaknesses* Users would need online access .	Ask, Investigate, Deprescribe
**Consultations and Training**		
Referral-based: Healthcare providers refer cases to a consult service with a physician specialist or pharmacist to help identify and manage potential prescribing cascade	*Strengths* Can consider integrating into existing services e.g. online specialist consult services (e.g., GeriMedRiska or eConsult Ontariob). Positive side effects if consultants provided explanation with consult notes. *Weaknesses* Difficulty is that healthcare providers would need to suspect a problem relating to prescribing cascades before making a referral, which requires the referring healthcare provider to know how to do these assessments. It is unlikely that this expertise already exists. Referring healthcare providers may need to provide an extensive history.	Ask, Investigate, Deprescribe
Quality Improvement (QI) Collaborative could involve a coach or academic detailing service to help teams identify and manage prescribing cascades	*Strengths* Can be facilitated by other QI collaboratives (e.g., primary care team quality improvement. collaboratives, SPIDERc) or Colleges (pharmacy, medicine, nursing) but would need a partnership. *Weaknesses* Needs to be built in as part of a team’s activities due to time commitment involved. Needs coach, process and infrastructure; intense from a logistical standpoint. This would also need learning and curriculum to ‘train the trainer’, ongoing meetings, etc. Requires lots of resources and would be expensive.	Ask, Investigate, Deprescribe
Practitioner/resource (e.g., consultant pharmacist) to help ‘flag’ people who are experiencing symptoms that could be drug-related	*Strengths* Positive spillover as repetitive identification may change behaviour, reduce need for specialist consultations. *Weaknesses* This would need to be accompanied by a thought process. Funding for research, design and marketing would be needed to do this well. Equity will vary based on “who” is delivering the service i.e., not all patients / prescribers have access to a consulting pharmacist.	Ask
**Knowledge Mobilization**		
Develop a knowledge mobilization and marketing campaign to help healthcare providers ask and verify if symptoms could be caused by a drug, and if so, how to manage them to identify or avoid a prescribing cascade.	*Strengths* Acceptable and practicable. *Weaknesses* Funding required. Challenging to measure effectiveness re: impact on patient important outcomes. Could consider this intervention to accompany others that focus on healthcare provider education.	Ask, Investigate, Deprescribe

^a^GeriMedRisk: www.gerimedrisk.com, ^b^eConsult Ontario: https://econsultontario.ca, ^c^SPIDER: Structured Process Informed by Data, Evidence and Research: www.spiderdeprescribing.com

## Discussion

The BCW process has been used by others to design interventions to improve medication management for patients experiencing multimorbidity [17], address polypharmacy [28], and support deprescribing [17–20, 28]. Our application to prescribing cascades is novel. Using the BCW process, and drawing from our past interviews, we identified potential behaviours for both healthcare providers and the public to address prescribing cascades. The team then selected the HCP ‘mega’ behaviour, A-I-D (ask, investigate, deprescribe), for intervention development. Psychological capability and physical opportunity were prioritized by our team as the highest priority behavioural drivers, followed closely by social opportunity and reflective motivation. This information was used to create 10 potential interventions to address prescribing cascades that can be further developed as single interventions or components of complex interventions.

Reasonable next steps for intervention development depend on the example selected. Broadly speaking, stakeholder input about prioritization and preferences, developing partnerships to co-design intervention materials, and designing feasibility studies are sensible next steps [14]. Our initial plan was to conduct a series of focus groups with stakeholders to prioritize and refine the intervention examples. However, as we planned the focus groups, we had two reflections. First, some of the proposed interventions focus on assisting healthcare providers to identify prescribing cascades (e.g., electronic alerts), not investigate or manage them. While these interventions may be helpful for preventing cascades, we were concerned that an intervention that helps to identify existing cascades is incomplete without also helping people to investigate and manage cascades. Second, when trying to develop more robust description of intervention examples, we realized that there is a necessary first step to intervention development. All potential interventions require the development of guidance for healthcare providers about how to perform the target behaviours necessary to ask, investigate and manage prescribing cascades (i.e., A-I-D).

Given the current state of evidence regarding the harms associated with cascades and the lack of intervention studies from which evidence-based recommendations could be drawn, efforts to develop an evidence-based clinical practice guideline would be premature. Instead, we suggest that a practice guidance tool is needed, which provides organized and systematically developed statements that help the healthcare provider make decisions about how to best care for a patient [29]. Once such a practice guidance tool is created, the interventions we proposed can be thought of as implementation or knowledge mobilization approaches (e.g., education initiatives, quality improvement collaboratives, health promotion campaigns, and consultant programs ([within the team or referral based]).

Our experience applying the BCW to design an intervention to address prescribing cascades was complex and contextually situated like the phenomena of prescribing cascades themselves. Although we present the application of BCW in a linear process, it was iterative, consistent with Medical Research Council (MRC) guidance regarding intervention development which outlines the process as “dynamic, iterative, creative, open to change and looking towards an evaluation process” [14]. As an example, we grappled with the level of granularity we applied as we specified our target behaviours including whether to call A-I-D a multistep behaviour or three distinct behaviours. From previous interviews [9, 10], these behaviours are intertwined. Investigating a potential prescribing cascade often involved managing it (i.e., deprescribing the medication potentially causing the side effect) and assessing the outcome, especially in cases where past medication history (e.g., reasons for the medications and timing) was unclear.

Our theory-guided process for intervention development to support addressing prescribing cascades has several strengths. The BCW process was applied by our diverse team of experienced scientists who are also healthcare providers. We were able to draw from our knowledge about concurrent, ongoing or evaluated health system initiatives focused on appropriate medication use and deprescribing which strengthens the feasibility of the interventions proposed. With respect to limitations, applying the BCW framework required several seemingly subjective judgements from our investigator team. This is mitigated by our efforts to transparently report these decisions in this paper. Our research team consisted of pharmacists and nurses, some, but not all, of whom have practiced as members of primary care interprofessional teams. Notably, our team did not include a physician, who are key ‘actors’ in the behaviour on which we focused. Thus, further consultation is required to fully represent physician perspectives in future stakeholder consultations.

Our next steps are to continue intervention development by gathering stakeholders’ feedback to understand the important features of a practice guidance tool that can be used by interprofessional primary care teams, as well as to explore how to maximize the reach and adoption of such a guidance tool in practice. Features include determining whether this will be an explicit (individual cascade-specific guidance) or an implicit tool (thought process), and intended users’ preferences for content, structure, layout, and ways of integration into daily care processes. As well, any practice guidance tool and intervention developed must specifically address psychological capability and physical opportunity given their importance as drivers of behaviour. A practice guidance tool and intervention(s) that supports health professionals’ psychological capability could include teaching them about what cascades are, provide common examples, and also discuss why and how to identify, investigate, and deprescribe them. To address physical opportunity, an intervention would ideally minimize barriers to data sharing across professionals (as part of an interprofessional team) and likely map out a workflow within the team for identifying potential cascades, investigating, and deprescribing them as well as monitoring the impacts of deprescribing efforts on patients.

## Conclusions

Prescribing cascades are an important and underrecognized contributor to polypharmacy and medication-related harm, and, therefore, a crucial target for intervention. We applied the BCW process to guide the design of intervention options that will support primary care healthcare providers practicing in interprofessional teams to address prescribing cascades in practice. When prioritizing interventions for future stakeholder consultation, we determined that the development of a practice guidance tool (i.e., which assists with identifying, investigating, and managing prescribing cascades) underpinned all the proposed interventions for tackling prescribing cascades in practice. Further research is needed to determine what primary care healthcare providers would need in this practice guidance tool and how it will be used in practice, to support its development.

## Supplementary Information

Supplementary Material 1: Table 1: Prioritization of Target Behaviours. Table 2: AACTT Framework for identifying target behaviours. Table 3: COM-B component analysis for the behaviour across all professions. Table 4: APEASE assessments for identified BCW Policy-Level options. Table 5: Potentially relevant behaviour change techniques and interventions. Table 6: Initial list of Intervention Examples.

## Data Availability

All data generated or analysed during this study are included in this published article and its accompanying supplemental information files.

## Abbreviations

AACTT: Action, actor, context, time, target
ADWE: Adverse drug withdrawal events
A-I-D: Ask, investigate, deprescribe
APEASE criteria: Acceptability, practicability, effectiveness / cost-effectiveness, affordability, side effects, equity
BCT: Behaviour change technique
BCW: Behaviour change wheel
COM-B Model: Capability, opportunity, motivation – behaviour model
HCP: Healthcare provider
MRC: Medical research council
