# Supplementary material for: A-I-D for cascades: an application of the Behaviour Change Wheel to design a theory-based intervention for addressing prescribing cascades in primary care
Source: Implement Sci Commun. 2024 Dec 5;5:137. doi: 10.1186/s43058-024-00673-x (PMC11619126; doi:10.1186/s43058-024-00673-x)
Supplement: Supplementary file 1 — Supplementary Material 1: Table 1: Prioritization of Target Behaviours. Table 2: AACTT Framework for identifying target behaviours. Table 3: COM-B component analysis for the behaviour across all professions. Table 4: APEASE assessments for identified BCW Policy-Level options. Table 5: Potentially relevant behaviour change techniques and interventions. Table 6: Initial list of Intervention Examples. [file 43058_2024_673_MOESM1_ESM.docx]

Title: A-I-D for Cascades: Designing a theory-based intervention for addressing prescribing cascades in primary care

Supplementary Appendix

Table 1: Prioritization of Target Behaviours

Table 2: AACTT Framework for identifying target behaviours

Table 3: COM-B component analysis for the behaviour across all professions

Table 4: APEASE assessments for identified BCW Policy-Level options

Table 5. Potentially relevant behaviour change techniques and interventions

Table 6. Initial list of Intervention Examples

**SUPPLEMENTARY MATERIALS**

**Table 1:** **Prioritization of Target Behaviours**

| **Behaviour** | **Impact of Behaviour Change** | **Ease of implementation** | **Potential for Spillover** | **Ease of Measurement** | **Global Prioritization** |
| --- | --- | --- | --- | --- | --- |
| Healthcare providers ask if patients’ signs and symptoms could be caused by a drug before prescribing a medication. | Very promising | Promising | Very Promising | Very Promising | Promising Identifying patients’ potential cascades is a necessary first step for addressing them. |
| Healthcare providers investigate and manage prescribing cascades before referral to specialists. | Very Promising | Promising | Very promising | Less Promising | Promising  Since investigate/manage is necessary to address prescribing cascades, opted to start here. |
| Patients ask if signs and symptoms could be caused by a drug. | Promising | Promising | Promising | Less Promising | Less Promising |
| Patients document and share medication information, history and experiences. | Promising | Less Promising | Very Promising | Promising | Less Promising |

***Notes:*** All factors were rated using unacceptable, less promising, promising, and very promising.

**Table 2:** **AACTT Framework for identifying target behaviours**

| **ACTION**  Behaviour that needs to change in terms that can be observed or measured | **ACTOR**  Each person/people that could do each of actions targeted) | **CONTEXT**  Physical location, emotional context, social setting in which action is performed | **TARGET**  Person/people for whom action is performed) | **TIME**  When action is performed i.e., date/time/frequency |
| --- | --- | --- | --- | --- |
| When they encounter a new sign/symptom or a possible prescribing cascade, healthcare providers perform the following: | | | | |
| **ASK** Ask/assess if the sign/symptom can be caused by one or more of the drugs the patient is taking | Physicians  Nurse Practitioners  Registered Nurse (assessment function with no prescriptive authority at present, may be limited prescribing to come)  Pharmacists | Interprofessional teams (vs. professionally siloed practices) in primary care and ambulatory care (i.e., specialty clinics where people receive care and return home in the same day) settings.  We decided to exclude hospital units and long-term care at this time due to variable involvement by physicians, pharmacists in these settings. | Patients | When patients present with new signs/symptoms, before ordering testing, before referral to specialists; during medication reviews |
| **INVESTIGATE** Investigate the sequence of events and reasons for medications | Physicians  Nurse Practitioners  Pharmacists |  |  | When a potential cascade is identified. |
| **DEPRESCRIBE**  A. To prevent a cascade or manage an existing one, there is a need to decrease, pause or stop potentially causative drug(s) (drug A) and monitor for adverse drug withdrawal events.  B. Then, if the cascade exists, there is a need to decrease, pause or stop drug B and monitor for adverse drug withdrawal events. | Physicians  Nurse Practitioners  Pharmacists |  |  | When a potential cascade is identified or confirmed. |

**Table 3: COM-B component analysis for the behaviour across all professions**

| **COM-B Component** | **Physician** | **Nurse practitioners** | **Registered Nurses** | **Pharmacists** |
| --- | --- | --- | --- | --- |
| Physical capability | - | - | - | - |
| Psychological capability | Y | Y | Y* | Y |
| Physical opportunity | Y | Y | Y | Y |
| Social opportunity | Y | Y | Y | Y |
| Reflective motivation | Y | Y | Y | Y |
| Automatic motivation | - | - | - | - |

*Different knowledge and skills compared with other professions.

**Table 4: APEASE assessments for identified BCW Policy-Level options**

| **Policy Option** | **Link to Intervention Type** | **APEASE summary** | **Retain for Intervention Development** |
| --- | --- | --- | --- |
| Guidelines | Education  Training  Environmental restructuring Enablement Restriction | Acceptability debated as there is no robust evidence base from which we can draw an ideal behaviour focused on investigation and management. An alternative would be to create a “good practice guideline” consensus guideline using Delphi etc. (Note: distinct from evidence-based guideline). | Yes |
| Communications and marketing | Education | Satisfies APEASE criteria. Many ways this could be delivered; however, it is recognized the content must first be developed. | Yes |
| Service provision | Education  Training  Enablement | Two ways this can be conceptualized:   1. People can provide the service of education where they teach others how to do this themselves in their practice (e.g., coach, academic detailing) 2. Consult model where provider refers to a service who performs the behaviour (or parts of it) for them (e.g., akin to they do right now with specialist referral)   Both options deemed to satisfy the APEASE criteria. | Yes |
| Environmental and social planning | Environmental  restructuring  Enablement | Deemed less relevant by APEASE criteria due to questionable effectiveness. (This policy category is “effective when the focus is not so much on changing people but changing the physical and social environment they inhabit”). | No |
| Legislation | Education  Training Environmental restructuring Enablement Restriction | There are not many legislated requirements related to the act of prescribing except as it pertains to narcotics and controlled drugs. Deemed to not be acceptable nor practicable. | No |
| Regulation | Education  Training Environmental restructuring Enablement Restriction | This could be incorporated into regulations (e.g., for renewals of licenses, as part of quality improvement plans for organizations), however, not acceptable/practical given evidence to date (i.e., lack of evidence showing the behaviour impacts outcomes). | No |
| Fiscal measures | Environmental restructuring Enablement | Not acceptable right now as there is a lack of evidence demonstrating behaviour impacts outcomes and there is no common practice upon which to base these measures. | No |

**Table 5. Potentially relevant behaviour change techniques and interventions**

| **Intervention Type** | **COM-B Component** | **Most Frequently Used BCT Label and Definition** | **Intervention Example: How BCT could be delivered** |
| --- | --- | --- | --- |
| Education | Psychological capability  Reflective motivation* (rated less important) | Information about social and environmental consequences (5.3) – provide information (e.g., written, verbal, visual) about social and environmental consequences of performing the behaviour (e.g., tell family physician about financial remuneration for conducting health screening) | e.g., explain that identifying and managing cascades can lead to improved health system resource use (by reducing polypharmacy, reducing downstream costs). This could be done as part of a best practice guideline, part of an educational program, or part of promotional materials for a consultant-based service. |
|  |  | Information about health consequences (5.1) – provide information (e.g., written, verbal, visual) about health consequences of performing the behaviour | e.g., missing prescribing cascades can lead to inappropriate polypharmacy; identifying and managing prescribing cascades can lead to improved health for patients (by reducing polypharmacy, pill burden, referral wait time, symptoms). This could be done as part of a best practice guideline, educational program, or promotional materials. |
|  |  | Feedback on behaviour (2.2) – monitor and provide information or evaluative feedback on performance of the behaviour (e.g., form, frequency, duration, intensity) | e.g., inform healthcare provider how many times they identified or managed cascades over a specific time period (e.g., in a day). Coach/practice observe to review prescribing over a time frame to identify potential cascades.   Audit and feedback interventions |
|  |  | Feedback on outcomes of behaviour (2.7) – monitor and provide feedback on outcome of performance of behaviour | e.g., inform the healthcare provider how many medications they deprescribed as result of identifying/ managing cascades; how many potential adverse events they avoided by identifying/ managing cascades |
|  |  | Prompts/cues (7.1) – introduce or define environmental or social stimulus with the purpose of prompting or cueing the behaviour. The prompt/cue would normally occur at time or place of performance | e.g., an electronic prompt or a sticker to remind the healthcare provider to ask, investigate, deprescribe, or remind healthcare providers to consider drug-related causes for most common side effects.  e.g., incorporate a prompt / flag into primary care provider electronic referral process ‘have you determined this issue is unlikely to be caused by a current medication the patient is taking?’ and referral can only proceed if tick yes |
|  |  | Self-monitoring of behaviour (2.3) – establish a method for person to monitor and record their behaviour as part of a behaviour change strategy | e.g., ask the healthcare provider to record whether they applied A-I-D, i.e., screened each patient’s medications for potential cascades, investigated, and deprescribed as appropriate  e.g., ask the healthcare provider to produce a report of their specialist referrals and record how often they investigate/manage a prescribing cascade before referral and look for change over time. |
| Training | Psychological capability  Physical opportunity | Demonstration of the behaviour (6.1) – provide an observable sample of the performance of the behaviour, directly in person or indirectly (via film, pictures) for person to aspire or imitate | e.g., demonstrate to healthcare providers how to identify/manage prescribing cascades as they take a patient history. This could be done through video or role play exercise.  video vignettes, online learning modules, |
|  |  | Instruction on how to perform a behaviour (4.1) – advise or agree on how to perform the behaviour | e.g., advise the healthcare provider about steps for identifying a cascade, how to manage a cascade by monitoring outcomes of deprescribing/pause and monitoring trial. This could be a video or interactive exercise. |
|  |  | Feedback on the behaviour (2.2) – monitor and provide information or evaluative feedback on performance of the behaviour (e.g., form, frequency, duration, intensity) (e.g., inform healthcare provider how many times they identified or managed cascades in a day) | ALSO IN EDUCATION |
|  |  | Feedback on the outcome(s) of behaviour (2.7) – monitor and provide feedback on outcome of performance of behaviour | ALSO IN EDUCATION |
|  |  | Self-monitoring of behaviour (2.3) – establish a method for person to monitor and record their behaviour as part of a behaviour change strategy | ALSO IN EDUCATION |
|  |  | Behavioural practice/rehearsal (8.1) – prompt practice or rehearsal of the performance of the behaviour one or more times in a context or at a time when the performance may not be necessary in order to increase habit and skill | e.g., prompt healthcare providers to practice identifying cascades on mock patients (could give them case studies to work through as part of a workshop/simulated or virtual patients). Examples include both prescription and non-prescription medications; also include perspectives from specialists. |
| Environmental Restructuring | Physical opportunity | Adding objects to the environment (12.5) – add objects to the environment in order to facilitate performance of the behaviour (e.g. provide free condoms to facilitate safe sex, provide nice toothbrush to improve toothbrushing technique) | e.g., a poster or other promotional materials that feature ‘Could this be caused by a drug?’ +/- link to a ‘Could this be caused by a drug?’ website) or ‘Before you refer, has this been caused by a drug?’ |
|  |  | Prompts/cues (7.1) – introduce or define environmental or social stimulus with the purpose of prompting or cueing the behaviour. The prompt/cue would normally occur at time or place of performance | ALSO IN EDUCATION |
|  |  | Restructuring the physical environment (12.1) – change or advise to change the physical environment in order to facilitate performance of the wanted behaviour or create barriers to the unwanted behaviour e.g., keep the cookie in a hard-to-reach cupboard | e.g., make requisition for a particular investigative test or referral form for specialist harder to access as a way to remind the healthcare provider to consider medication-related cause for symptom (where they would normally first do the test or refer onward)  e.g., questionnaire for patients to report before visit with a healthcare provider (e.g., in the waiting room or emailed in advance) |
| Enablement | Psychological capability  Physical opportunity | Social support (unspecified) (3.1) – advise on, arrange or provide social support (e.g., from friends, relatives, colleagues etc.) or non-contingent praise or reward of the performance of the behaviour, e.g., advise person to call a buddy when want a cigarette | e.g., listserv, journal club, quality improvement collaborative, blog with regular discussion of successes and challenges in identifying and managing prescribing cascades; consider testimonials from specialists indicating when appropriate to investigate and manage prescribing cascades before specialist referral. |
|  |  | Social support (practical) (3.2) – advise on, arrange or provide practical help (from friends, relatives, etc.) for performance of the behaviour, e.g., ask the partner of a patient to put a pill out so patient takes it | e.g., listserv, journal club, quality improvement collaboratives, blog with regular discussion of successes and challenges in identifying and managing prescribing cascades; consider testimonials from specialists indicating when appropriate to investigate and manage prescribing cascades before specialist referral  e.g., phone a friend (e.g., pharmacist) when you want help.  e.g., have a pharmacist or other resource available to help ‘flag’ people who are experience symptoms that could be drug-related |
|  |  | Goal setting (behaviour) (1.1) – set or agree on a goal defined in terms of behaviour to be achieved | e.g., set a target to investigate and manage medication-related causes for a specific number of patients (before referral to specialists) within a certain time frame |
|  |  | Goal setting (outcome) (1.3) – set or agree on a goal defined in terms of a positive outcome of wanted behaviour e.g., lose 0.5 kg over one week as an outcome of changed eating patterns | e.g., set a target to deprescribe (trial, pause, monitor) one medication resulting from a potential cascade for a specific number of patients (before referral to specialists) within a certain time frame |
|  |  | Adding objects to the environment (12.5) – add objects to the environment in order to facilitate performance of the behaviour (e.g., provide free condoms to facilitate safe sex, provide nice toothbrush to improve toothbrushing technique) | IN ENVIRONMENTAL RESTRUCTURING |
|  |  | Problem solving (1.2) – analyze or prompt the person to analyse, factors influencing the behaviour and generate or select strategies that include overcoming barriers and/or increasing facilitators | e.g., provide an academic detailing or practice facilitator interaction in which healthcare providers can work through case studies designed to help them practice the investigation and management clinical thought process; examples to include both prescription and non-prescription medications; also include perspectives from specialists. Ask healthcare providers to self-reflect on a recent case where they prescribed a cascade, describe their thought process at the time, and generate strategies to address it. |
|  |  | Action planning (1.4) – prompt detailed planning of performance of the behaviour (must include at least one of context, frequency, duration, intensity). Context can be environmental or internal | e.g. provide an academic detailing or practice facilitator interaction in which healthcare providers create SMART plans to investigate if patients are experiencing prescribing cascades, and if so, to manage them to resolution. |
|  |  | Self-monitoring of the behaviour (2.3) – establish a method for person to monitor and record their behaviour as part of a behaviour change strategy | e.g., ask the healthcare provider to record whether they screened each patient’s medications for potential prescribing cascades. This could be done through a tracking sheet or log or mobile app.  e.g. ask the healthcare provider to produce a report of their specialist referrals and record how often they investigate/manage a prescribing cascade before specialist referral (e.g. never, sometimes, usually, always) and look for change over time.  e.g. ask pharmacist to produce a report of their patient interactions and record how often they investigate/manage a prescribing cascade before filling a prescription, recommending a product, or referring to the primary care provider (e.g. never, sometimes, usually, always) and look for change over time |
|  |  | Restructuring the physical environment (12.1) – change or advise to change the physical environment in order to facilitate performance of the wanted behaviour or create barriers to the unwanted behaviour e.g., keep the cookie in a hard-to-reach cupboard | IN ENVIRONMENTAL RESTRUCTURING |
|  |  | Review behaviour goal(s) (1.5) – review behaviour goals jointly with the person and consider modifying goal(s) or behaviour change strategy in light of achievement | e.g., provide an academic detailing or practice facilitator interaction in which the detailer/facilitator reviews the healthcare provider’s SMART plans (to investigate if patients are experiencing prescribing cascades, and if so, to manage them to resolution) and helps them modify as needed. |
|  |  | Review outcome goal(s) (1.7) – review outcome goals jointly with the person and consider modifying in light of achievement | e.g., provide an academic detailing or practice facilitator interaction in which the detailer/facilitator reviews the healthcare provider’s SMART plans (e.g. in terms of deprescribing) and helps them modify as needed in light of their outcome goals. |

**Table 6. Initial list of Intervention Examples**

| **Intervention** | **Intervention Prioritized for Future Stakeholder Consultation** |
| --- | --- |
| **Online learning module** for healthcare providers that teaches them to identify and manage prescribing cascades & set personal ‘SMART’ goals | Yes |
| **Web-based or other e-tool related** that help healthcare providers identify potential prescribing cascades **e.g. thought process or algorithm** | No. The work to create these is considerable and expensive. Further, one cannot create an e-tool without first creating the thought process as an initial first step. |
| **Web or other e-tool related to list of prescribing cascades** that help healthcare providers identify potential prescribing cascades **e.g. analyzes list** | No. The work to create these is considerable and expensive. If this is pursued, may need to prioritize development efforts (e.g., focus on drugs known to contribute to falls, urinary incontinence, other geriatrics syndromes). It would not be feasible to attempt to include all side effects for all medications. |
| **Addition of box on referral form** for specialist consult to trigger healthcare providers to ask if problem likely to be caused by a drug | No. More difficult given the myriad of forms available, would be challenging to implement on a system level. |
| **Consult service (referral based)** to help identify and manage prescribing cascades | Yes |
| **Consult service (coach / academic detailing)** to help identify and manage prescribing cascades | Yes |
| **Good practice guidelines** for prescribing cascades | Yes |
| Incorporate learning about prescribing cascades into **healthcare provider undergraduate education** | No. This is not an action in and of itself. Also, the team decided to focus on currently practicing healthcare providers for future focus groups re: practice guidance tool. |
| **Audit and feedback** for individual healthcare providers that assesses their performance in identifying and managing prescribing cascades  **e.g.,** report that assesses target behaviour (assessing / deprescribing) or outcome (avoiding potential adverse drug events). Could use a tracking sheet, or post-visit survey or could target a number of patients, specific symptoms, certain time frame, or referral to specialists. Can provide individualized feedback. | No. Audit and feedback program without a robust evidence base to demonstrate that addressing cascades improves patient outcomes seems premature given the resource intensity involved. |
| **Have pharmacist / resource available to help ‘flag’ people** who are experiencing symptoms that could be drug-related | Yes |
| **Listserv, journal club, or quality improvement collaborative** to stimulate discussion amongst healthcare providers about prescribing cascades | Yes |
| **Demonstrate clinical thought process** of investigating and managing prescribing cascade for healthcare providers | Yes |
| **Questionnaire for patients to fill out before visit** with a healthcare provider: ‘have you started any new drugs recently?’  e.g. questionnaire done in waiting room, email in advance or through telephone / online booking | No. This behaviour requires patients to complete the questionnaire. Acceptability by healthcare providers is less clear. |
| **Electronic prompt / sticker** to remind healthcare providers to ask, investigate, deprescribe (not necessarily triggered by new prescription)  E.g. need to click yes ‘I have attempted to identify and manage prescribing cascades that could be contributing or clouding the issue’ (can only proceed if they tick ‘yes’) and the ‘prescribing cascades’ words could directly link to a website / resource (links to web or e-tool medication list intervention) | No. Electronic prompts without additional support about how to investigate and manage prescribing cascades is unlikely to change practice. First step would be to develop a process for healthcare providers to follow for addressing prescribing cascades. |
| **Electronic alert at the point of prescribing** to remind healthcare provider to ask, investigate, deprescribe (triggered by a new prescription) | No. Electronic prompts without additional support about how to investigate and manage prescribing cascades is unlikely to change practice. First step would be to develop a process for healthcare providers to follow for addressing prescribing cascades. |
| **Marketing / increasing awareness** of prescribing cascades | Yes |
